# Graph-based homogenisation for modelling cardiac fibrosis

**DOI:** 10.1016/j.jcp.2022.111126

**Published:** 2022-06-15

**Authors:** Megan E. Farquhar, Kevin Burrage, Rodrigo Weber Dos Santos, Alfonso Bueno-Orovio, Brodie A.J. Lawson

**Affiliations:** a Australian Research Council Centre of Excellence for Mathematical and Statistical Frontiers, School of Mathematical Sciences, Queensland University of Technology, Brisbane, Australia; b Department of Computer Science, Oxford University, Oxford, United Kingdom; c Department of Computer Science and Program on Computational Modeling, Universidade Federal de Juiz de Fora, Juiz de Fora, Brazil; d Centre for Data Science, Queensland University of Technology, Brisbane, Australia

**Keywords:** Cardiac modelling, Homogenisation, Graph-based modelling, Eikonal methods, Numerical upscaling

## Abstract

Fibrosis, the excess of extracellular matrix, can affect, and even block, propagation of action potential in cardiac tissue. This can result in deleterious effects on heart function, but the nature and severity of these effects depend strongly on the localisation of fibrosis and its by-products in cardiac tissue, such as collagen scar formation. Computer simulation is an important means of understanding the complex effects of fibrosis on activation patterns in the heart, but concerns of computational cost place restrictions on the spatial resolution of these simulations. In this work, we present a novel numerical homogenisation technique that uses both Eikonal and graph approaches to allow fine-scale heterogeneities in conductivity to be incorporated into a coarser mesh. Homogenisation achieves this by deriving effective conductivity tensors so that a coarser mesh can then be used for numerical simulation. By taking a graph-based approach, our homogenisation technique functions naturally on irregular grids and does not rely upon any assumptions of periodicity, even implicitly. We present results of action potential propagation through fibrotic tissue in two dimensions that show the graph-based homogenisation technique is an accurate and effective way to capture fine-scale domain information on coarser meshes in the context of sharp-fronted travelling waves of activation. As test problems, we consider excitation propagation in tissue with diffuse fibrosis and through a tunnel-like structure designed to test homogenisation, interaction of an excitation wave with a scar region, and functional re-entry.

## Highlights

• Novel numerical method for capturing fine-scale heterogeneities using a coarse mesh.
• Competitive homogenisation technique that functions naturally on irregular grids.
• Error from Graph-based method is consistent with upscaling the homogeneous problem.
• Performs well on some challenging problems of interest in Cardiac electrophysiology.

## 1 Introduction

Cardiovascular diseases are the leading causes of death, accounting for 30% of deaths worldwide. Understanding the mechanisms and processes that occur in the heart is very important for advancing diagnosis and treatment tools for individual patients. This has led to an increasing interest in the literature for so-called patient specific modelling, where a computational model for the cardiac electrophysiology of an individual patient is constructed by considering their unique anatomical, physiological and/or genetic features [1], [2]. These models feature complex descriptions of ion channel dynamics on the cellular level [3], [4], [5], [6], complex spatiotemporal patterns of activation [7], [8], [9] and spatial modelling incorporating underlying tissue mechanics [10], [11], [12].

Computational modelling plays an important role in understanding the impacts that cardiac fibrosis has on the propagation of electrical signals in the heart. They can be used to provide predictions to inform clinical practise [13], [14]. In the move to patient specific modelling, reduced models have been used in the context of cardiac modelling through techniques such as, homogenisation [10] and graph-based methods [15], [16], [17].

In this work we develop a novel graph-based homogenisation method that is based around Eikonal-type methods that are popular for rapid computation of wavefront propagation [18] and in cardiac modelling [15]. However, while the standard Eikonal approach calculates activation times for cardiac models, we use the Eikonal theory to capture the impacts of micro-scale tissue properties in standard monodomain simulations that feature the full suite of electrophysiological dynamics. Importantly, our novel concept of using graph-based information to determine the effective properties of tissue relies on none of the assumptions made by traditional homogenisation approaches. We present numerical results of activation potential propagation through fibrotic tissue that show the method successfully models micro-scale behaviour on a macro-scale mesh. We show that the method produces fast and accurate results when compared to a micro-scale reference solution, and that it does not produce any significant error. While the methods presented in this work are presented in terms of their effectiveness in the context of cardiac fibrosis, they can be applied in other travelling wave applications where the conductivity or diffusion tensor is varying in space. This could include the application of the methods to modelling the effects of recent treatment advances for treating ischemic cardiac injury, namely, injectable hydrogels [19], [20], [21], [22].

We begin in Section 2 by presenting a background of homogenisation approaches in cardiac modelling. Then in Section 3 we present a graph-based homogenisation method and the basis on which it was developed. We present some numerical test problems in Section 4 to show the effectiveness of using the graph-based homogenisation approach on a variety of cardiac electrophysiology problems: including the propagation of electrical impulses through tissue with diffuse fibrosis, a tunnel-like domain, and tissue featuring a region of scarring. We also apply our homogenisation to a spiral wave re-entry. In Section 5 we discuss the performance of our graph-based homogenisation and present conclusions in Section 6.

## 2 Background

Fibrosis is the excess of extracellular matrix that affects many organs in the body, including the heart, lungs and liver. In the heart, fibrosis can occur due to, for example, heart disease, myocardial infarction, Chagas disease, inflammation, scarring and ageing [23], and has been shown to be a key trigger for common cardiac arrhythmias such as atrial fibrillation [13], [24], [25] and premature ventricular contractions [26]. Fibrosis blocks the electrical connectivity between neighbouring cells, slowing or even blocking the propagation of the electrical signal through the tissue. The severity of these effects in terms of the heart's electrical function is determined not just by the volume of fibrosis, but also by the type of pattern that it forms [26]. Further complicating matters, multiple patterns can be present within heart tissue simultaneously [23].

Computational modelling is an important component of understanding the impacts of fibrosis, owing to the complex interplay between heterogeneities in conductivity and the dynamic regulation of sarcolemmal ion flow by cardiac myocytes. Computer models enable mechanistic investigation of this interplay [11], and can even provide predictions with which to inform clinical practice [13], [14]. However, it is important to balance the accuracy of these models with computational efficiency. Detailed three-dimensional anatomically accurate models can often take days to run, even on large supercomputers, when simulating over multiple heart beats. While such an approach may be appropriate when the parameters for the models representing individual patients are known, it is a significant road block for determining the aspects that are specific to each patient. In order to move forward to large scale simulation, methods are focused on ways to reduce the computational costs, while retaining the accuracy of the results. This has motivated the use of reduced models in the cardiac context, obtained for example by homogenisation [10], graph-based methods [15], [16], [17], or statistical emulation [27].

Incorporating fibrosis in cardiac simulations, particularly on the microscopic length scale required to resolve its complex patterns of arrangement (∼ 10 μm) [23], puts further pressure on computational models. Indeed, the resolution of imaging techniques and issues of computational feasibility typically limit the resolution of anatomically accurate meshes to have minimum spacing of 100 micrometres [7], an order of magnitude too large. As such, inherent differences in the structure of the tissue at a micro scale are lost, despite the potential to dramatically affect the macroscopic behaviour, in a manner differing from model to model. Homogenisation presents a natural means of addressing this problem.

The basic principle of homogenisation is to modify a macroscopic model to include aspects of the microscopic structure. Numerical homogenisation uses techniques to compute the average behaviour of materials providing *effective* parameters for the diffusion, conductivity or viscosity tensors. Therefore, it becomes a natural way to address the mesh spacing limitations in cardiac models without needing to simulate on the fine-scale mesh. When the microscopic structure varies throughout the problem domain, the typical homogenisation approach is through block homogenisation [28]. This assumes the computational domain to be made up of periodically tiled microscopic subdomains, and uses each individual subdomain to compute the corresponding effective tensor of the material in the macroscopic model. Thus, homogenisation relies on predefined blocks and does not consider information outside each element.

There has been some exploration of the use of homogenisation in cardiac electrophysiology. In fact, the bidomain equation can be derived by homogenisation of the intra-cellular and extra-cellular domains [29], [30], [31], [32], [33]. However, when it comes to using homogenisation to represent cellular fibrosis it is generally limited to using spatially periodic structures [10], [34]. One work introduced arbitrarily arranged obstacles into larger-scale simulations and used black-box multigrid homogenisation [35]. There has also been some recent work using proper generalised decomposition methods [36] and data assimilation approaches [37], [38] for efficient estimation of cardiac conductivities.

Another approach to reducing computation time and increasing accuracy in cardiac electrophysiology is through the use of Eikonal or graph-type methods. The use of these methods in cardiac electrophysiology emerged in the 1990s [15], [16], [17]. The idea of these Eikonal type methods is to generate a graph on the domain of interest and use the time it takes to travel between the nodes of the graph to compute the activation map. As such, the influence of the reaction term is removed, and Eikonal approaches cannot naturally predict important dynamics such as re-entry and conduction block. Nevertheless, these methods have been used in the estimation of Purkinje tree pathways and the Purkinje-myocardial junctions [39], [40], [41]. An inverse Eikonal approach has also been implemented as a means of recovering three-dimensional activation sequences from epicardial activation maps [42]. A graph-based homogenisation approach was taken to move from a sub-cellular mesh to a discrete mesh [43]. A comparative study of graph path-finding algorithms and the similar fast marching approach was performed to validate the effectiveness of these approaches in comparison to the monodomain model [44].

Some extensions to the Eikonal approach in the cardiac electrophysiology context have also been proposed, seeking to rectify its obvious limitations. An Eikonal-diffusion method has been proposed to allow simulation of re-entrant activation patterns and wavefront collisions [45], and a reaction-Eikonal and reaction-diffusion-Eikonal approach have been used for efficient computation of electrograms using coarse mesh domains [46]. However, these approaches still require significant mesh refinement in order to represent obstacles and heterogeneities on the microscopic scale. Here, we pose a different question — how can the ideas underpinning graph-based methods be used to address the limitations of traditional homogenisation approaches, for the challenging models associated with computational electrophysiology?

## 3 Methods

### 3.1 The monodomain model

In line with many other works in cardiac electrophysiology, we use the monodomain equation to describe cardiac electrical propagation [8],

(1) $$\frac{\partial V_{m}}{\partial t}=\frac{\lambda }{\chi C_{m}(\lambda +1)}\nabla \cdot \left(\Sigma \nabla V_{m}\right)-\frac{1}{C_{m}}(I_{ion}(V_{m},\eta )+I_{stim})\frac{\partial \eta }{\partial t}=f(V_{m},\eta )0=(\Sigma \nabla V_{m})\cdot \overset{ˆ}{n}.$$

Here, Σ is the conductivity tensor, $V_{m}$ is the membrane potential, $I_{ion}$ is the ionic current, *χ* is the membrane surface-to-volume ratio, $C_{m}$ is the membrane capacitance, *λ* is the intracellular to extracellular conductivity ratio and $I_{stim}$ is the externally applied stimulus current. The ionic current model is given by ***f***, and in this work we consider the reduced ten Tusscher and Panfilov 2006 epicardial model [6]. The parameter values and units for equation (1) that are used in this work can be found in Table 1.

**Table 1:**
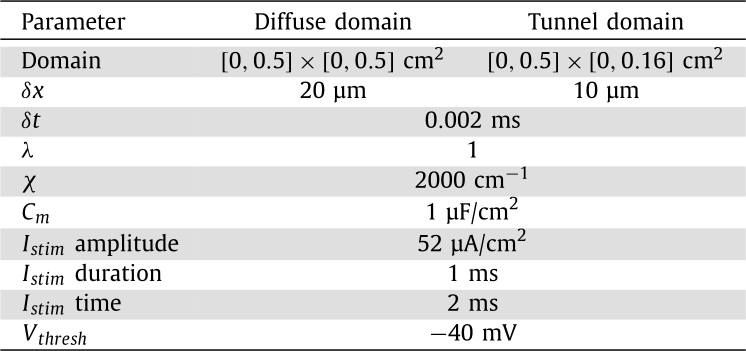
Model parameters.

### 3.2 Eikonal methods

Within cardiac modelling (and other applications with a moving wavefront), Eikonal methods have been used to describe the travel time of the wave front. The general Eikonal equation can be written as,

(2) $$F\sqrt{\nabla T^{T}\;\Sigma \;\nabla T}=1$$

where *F* is the speed function, ∇ is the gradient operator, *T* is the arrival time of the wave front as a function of the spatial location and Σ is the conductivity tensor.

One approach in the literature to solving equation (2) is to use a connected graph to describe the cardiac tissue [44], [47], [48], [49]. A graph is a set of nodes $\left\{n_{i}\right\}$, connected by a set of edges $\left\{e_{i,j}\right\}$. Each edge $e_{i,j}$ has an associated positive cost $C_{ij}$. Graphs can have directed costs, so that $C_{ij}\neq C_{ji}$, however, in this work, we consider only undirected graphs where $C_{ij}=C_{ji}$. To solve equation (2), the costs can be calculated according to

(3) $$C_{ij}=\sqrt{x_{ij}^{T}\Sigma^{-1}x_{ij}}/F$$

where $C_{ij}$ is the cost from node *i* to node *j* in the same cell, *F* is the speed function, Σ is the conductivity tensor representing the anisotropy of the tissue and $x_{ij}$ is the vector between nodes *i* and *j*.

**Fig. 1:**
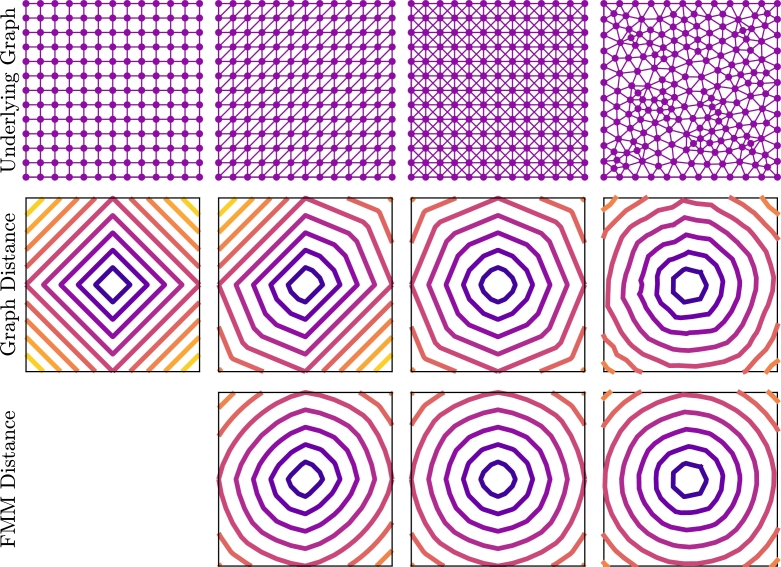
Comparison of graph methods and the Fast Marching Method (FMM) for calculating distance from the centre of the domain. The first row is the underlying graph that is used to compute the distances and the second and third rows are contours of the computed distances for a graph method and FMM, respectively.

**Fig. 2:**
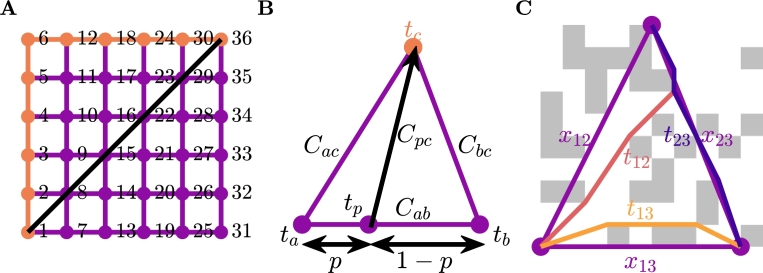
**A**: An example of standard graph shortest path algorithms failing to recover the physical shortest path. **B**: Visual depiction of FMM on a single element. This method interpolates the solution along the bottom edge to get *t*_*p*_ and finds the minimum value for *t*_*c*_ when crossing the element is allowed. **C**: An example of the graph-based homogenisation method on an individual element.

Standard graph-based methods such as $A^{⁎}$ and Dijkstra's algorithm can be used to compute activation time, but there are some issues with this approach [44], [47]. These methods begin at the start node and proceed by computing the activation time at the connected nodes by adding the cost along the edges of the graph, and progressively advance nodes until the desired node is reached. The Dijkstra algorithm advances nodes based on minimum activation time, whereas the $A^{⁎}$ algorithm uses a heuristic that gives preference to nodes in the direction of the end node. Considering these standard approaches, we are constrained to the edges of the graph, and, depending on the underlying mesh, this can have dramatic effects (Fig. 1). In the first row of this figure we depict four different graphs on a square unit cell domain. In the second row of the domain we depict the contours of distances from the node in the middle of the domain ($\Sigma =I_{2}$) calculated using standard graph methods. In these figures, we can see that the underlying graph has a significant effect on the computed distances, particularly for the structured meshes. The structural artefacts remain in the solution no matter how refined. The reason for this artefact can be seen in Fig. 2A. We can see here that the shortest path from $n_{1}$ to $n_{36}$ in this figure is to cross the diagonal, however, as we are forced to stay on the graph, this distance is incorrect by a factor of $\sqrt{2}$.

We can mitigate the effects of the underlying graph for triangular graphs, by using the Fast Marching Method (FMM) [44], [48], [49]. This method presents an approach that allows movement across elements and is standard in Eikonal approaches in cardiac modelling. A schematic showing this approach can be seen in Fig. 2B. The idea of the approach is to interpolate the solution across an edge between two known nodes and use triangle geometry to compute the cost from any point on that line. For the image in the figure, if $t_{a}$ and $t_{b}$ are known, then $t_{c}$ can be computed through a point $t_{p}$ with

(4) $$t_{c}=(t_{b}-t_{a})p+t_{a}+\sqrt{A{\left(p-H\right)}^{2}+K},$$

where

$$A=C_{ab}^{2},\;H=\frac{C_{ab}^{2}+C_{ac}^{2}-C_{bc}^{2}}{2C_{ab}^{2}},\;K=C_{ac}^{2}-AH^{2},\;p\in [0,1].$$

Using standard minimisation techniques, the value of *p* that minimises equation (4) is given by

(5) $$p^{⁎}=\min\left(1,\max\left(0,H-\frac{t_{b}-t_{a}}{A}\sqrt{\frac{K}{1-(t_{b}-t_{a})/A}}\right)\right).$$

FMM is implemented in a Dijkstra-like path finding algorithm, this can be seen in Algorithm 1, Algorithm 2. The Dijkstra-like path finding algorithm selects the node to advance based on the minimum unknown time. We could similarly implement FMM in a $A^{⁎}$-like path finding algorithm, that selects the unknown node to advance based on the minimum of a heuristic that is the sum of the time and the distance from the goal node. However, the effectiveness of this approach is reduced for graphs that have underlying anisotropy and is further reduced with fibrosis.

**Algorithm 1:**
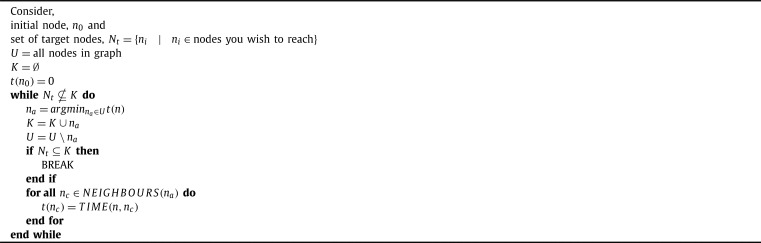
Generic Dijkstra-like Path finding.

**Algorithm 2:**
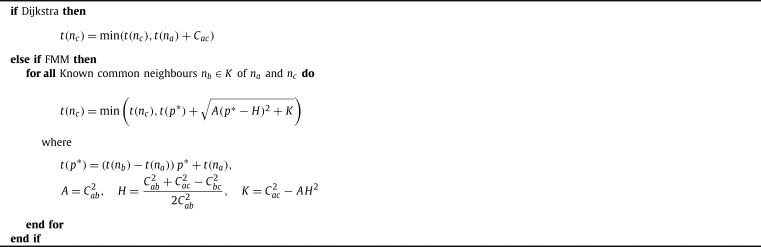
*TIME*(*n*_*a*_,*n*_*c*_).

The results of using FMM to compute the distance from the centre of the domain in our earlier example can be seen in the third row of Fig. 1. Here we can see that the underlying structure of the graph does not have as much of an effect as it did with standard graph methods. Refining the underlying mesh (not shown here) results in improved accuracy of the FMM distance, thus removing the effects of the underlying structure.

### 3.3 Novel graph-based homogenisation

We combine the graph-Eikonal approach and homogenisation techniques to develop a graph-based homogenisation approach. The basic idea of the approach is to use FMM (Algorithm 1, Algorithm 2) on the microscale graph to compute the time between connected nodes of a macroscale mesh, and use those times to compute effective diffusivities for each element.

The first step of the process is to create a graph from the microscopic domain. For the examples in this work, we use a grid of cells that can either be healthy or fibrotic. The fibrotic cells are considered to be non-conductive, and as such, the nodes in those cells are not connected. For the healthy cells, we consider connections between all corners, including across both diagonals. We compute the cost of each edge of the graph using equation (3) with F=1, $C_{ij}$ is the cost from node *i* to node *j* in the same cell, Σ is the conductivity tensor of the tissue and $x_{ij}$ is the vector between nodes *i* and *j*.

The second step is to determine the macroscale mesh. Unlike traditional homogenisation, we are free to select the nodes making up this mesh without concern for subproblem boundary conditions or notions of periodicity. However, there are two important considerations that we need to take into account in this process. First, the macroscopic mesh should still encompass the full microscale domain, and secondly the set of nodes selected should result in a mesh of sufficient numerical quality. To achieve this, we use a simulated annealing approach with a fixed temperature [50] for selecting the set of reduced nodes, randomly selecting a subset of the nodes and then repeatedly choosing a node inside the subset at random, and trialling the effects of replacing it with a random node not in the subset. These trials are accepted or rejected according to how they affect the quality of resulting mesh — see Algorithm 3 for more details regarding this process. To capture the full microscale domain, we use MATLABs alphaShape function to find the shape of the domain and then the boundaryFacets and polyshape functions to find all the corners of the shape required to form the shape of the domain and always include these nodes in the reduced set. Mesh quality is measured by a metric that includes the quality of the triangular elements, and the extent of variation in triangle area across the entire mesh,

(6) $$q=\frac{\min(t_{q})+\mathrm{mean}(t_{q})}{2}-\frac{0.075\;\mathrm{std}(\text{Area})}{\mathrm{mean}(\text{Area})},$$

where $t_{q}$ is the normalised quality of each triangular element measured by [51]

(7) $$t_{q}=\frac{4\sqrt{3}\mathrm{Area}}{\text{sum of squared side lengths}}.$$

The value 0.075 serves as a weighting to appropriately balance the two different metrics for mesh quality, and was selected heuristically. The triangle quality measure is important for numerical performance, however on its own it is not enough when there is only a small number of nodes in the macroscale mesh. This is especially a problem for the coarsest macroscale meshes that are in the third test problem, causing a mixture of large and small triangles resulting in elements too big for the signal to propagate through the domain.

**Algorithm 3:**
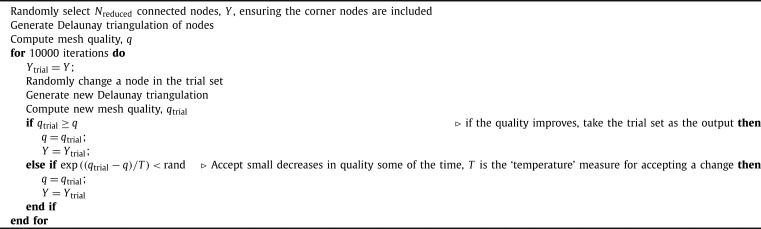
Macroscale mesh generation - output *Y*.

The next step in the graph homogenisation process is to use the microscale graph to generate a macroscale graph on the coarse scale mesh. For each element, we use FMM (Algorithm 1, Algorithm 2) to compute the shortest time between each of the nodes on the vertices as shown in Fig. 2C.

The final step of the process is to convert the macroscale graph into a partial differential equation. This step is done by determining an effective diffusivity for each element. For each element we know the time for each edge $t_{i}$, and we can determine the symmetric $\Sigma_{\mathrm{eff}}$ for each element by solving,

(8) $$t_{12}=\sqrt{x_{12}^{T}\Sigma_{\mathrm{eff}}^{-1}x_{12}}\;t_{13}=\sqrt{x_{13}^{T}\Sigma_{\mathrm{eff}}^{-1}x_{13}}\;t_{23}=\sqrt{x_{23}^{T}\Sigma_{\mathrm{eff}}^{-1}x_{23}}\;,$$

where again $x_{ij}$ is a vector between nodes *i* and *j* and $t_{ij}$ is the time between the nodes *i* and *j*. An example element can be seen in Fig. 2C. If there is no solution to (8), we assume there is no conductivity in that element. With effective conductivities for each element defined, we then have an homogenised version of equation (1),

(9) $$\frac{\partial V_{m}}{\partial t}=\frac{\lambda }{\chi C_{m}(\lambda +1)}\nabla \cdot \left(\Sigma_{\mathrm{eff}}(x)\nabla V_{m}\right)-\frac{1}{C_{m}}(I_{ion}(V_{m},\eta )+I_{stim})\frac{\partial \eta }{\partial t}=f(V_{m},\eta )0=(\Sigma_{\mathrm{eff}}(x)\nabla V_{m})\cdot \overset{ˆ}{n},$$

where the boundary conditions of the microscale problem extend naturally into the homogenised problem.

### 3.4 “Standard” homogenisation

To serve as a comparison for the graph-based methods demonstrated here, we also apply a more standard homogenisation approach recently brought to cardiac fibrosis [52] to our test problems. This approach uses the method of volume averaging [53] to derive effective conductivities for mesh elements on the larger scale, and results in a homogenised monodomain model slightly different to equation (9),

(10) $$\frac{\partial V_{m}}{\partial t}=\frac{\lambda }{\phi \chi C_{m}(\lambda +1)}\nabla \cdot \left(\phi \Sigma_{\mathrm{eff}}(x)\nabla V_{m}\right)-\frac{1}{C_{m}}(I_{ion}(V_{m},\eta )+I_{stim})\frac{\partial \eta }{\partial t}=f(V_{m},\eta )0=(\Sigma_{\mathrm{eff}}(x)\nabla V_{m})\cdot \overset{ˆ}{n}.$$

The difference is the presence of the ratio $\phi =|\Omega_{c}|/|\Omega |$ of the volume of the conductive portion of a macro-scale element $\left|\Omega_{c}\right|$ to the volume of the whole element, |Ω|. This is the volume fraction (or porosity), and is a spatially-varying quantity on the macroscopic scale on which equation (10) is defined.

For each separate macro-scale element, Ω, the effective conductivity is calculated by integrating the solutions to a set of corresponding closure problems, $w=(w_{x},w_{y})$, over the conductive portion of that element, $\Omega_{c}$. Specifically,

(11) $$\Sigma_{\mathrm{eff}}=\frac{1}{\left|\Omega_{c}\right|}\int_{\Omega_{c}}\Sigma \left(I+\nabla w^{T}\right)\;d\Omega_{c},$$

where $\left|\Omega_{c}\right|$ indicates the volume of the conductive portion of the element, ***I*** is the identity matrix and $\nabla w^{T}$ denotes the transpose of the Jacobian of ***w***.

In the implementation used here, closure subproblems are solved on a domain extended beyond Ω in order to reduce the effects of their boundary conditions upon their solution [54]. Denoting the extended domain $\Omega_{s}$ and its conductive portion $\Omega_{sc}$, the closure problems take the form

(12) $$\begin{array}{cccc} 0 & =\nabla \cdot (\Sigma \left(\nabla w_{i}+e_{i}\right)) & & \;\text{on }\Omega_{sc}\\ 0 & =w_{i} & & \;\text{on }\partial \Omega_{s}\\ 0 & =(\Sigma \left(\nabla w_{i}+e_{i}\right))\cdot \overset{ˆ}{n} & & \;\text{on }\partial \Omega_{sc}\setminus \partial \Omega_{s}\;\text{,} \end{array}\;\;\;\;i\in \{x,y\}$$

where $e_{i}$ are the standard basis vectors and $\overset{ˆ}{n}$ denotes the normal vector for boundaries with non-conductive material ($\partial \Omega_{sc}\setminus \partial \Omega_{s}$). This choice of boundary condition on the edges of the averaging volume is known as a “linear” boundary condition [55], the choice that proved most suitable in a series of cardiac electrophysiology test cases (results in a work under submission [52]).

Owing to the assumption of periodicity that the derivation of (11) relies upon [53], homogenisation approaches are typically constructed on regular quadrilateral grids that allow for natural implementation of different boundary conditions and consistent means of extending Ω to $\Omega_{s}$ [54], [55], [56]. Our implementation of standard homogenisation follows this approach, using square macro-scale elements, with closure problem domains extended by half their width in all directions. One of the advantages of our graph-based method for homogenisation is the lack of dependence on periodicity and the freedom to use irregular macro-scale meshes without needing to extend the basic approach.

### 3.5 Partial differential equation solutions

We now have two monodomain partial differential equations to solve, the micro-scale reference solution (1) and the homogenised macro-scale solutions (9). As mentioned in Section 3.1, we consider the ten Tusscher and Panfilov cell model [6] for the form of ***f*** and $I_{\mathrm{ion}}$. We solve equations (1) and (9) using Crank-Nicholson Adams-Bashforth time integration with a vertex-centred finite volume method, approximating integrals over control volumes by using the exact integration of linear interpolants of the nodal values. For the microscale problem (1) we have a quadrilateral mesh, and for the macroscale problem (9) we have a triangular mesh. We use the second-order generalisation of the Rush-Larsen method presented by [57] as our method of time-stepping.

## 4 Numerical tests

**Fig. 3:**
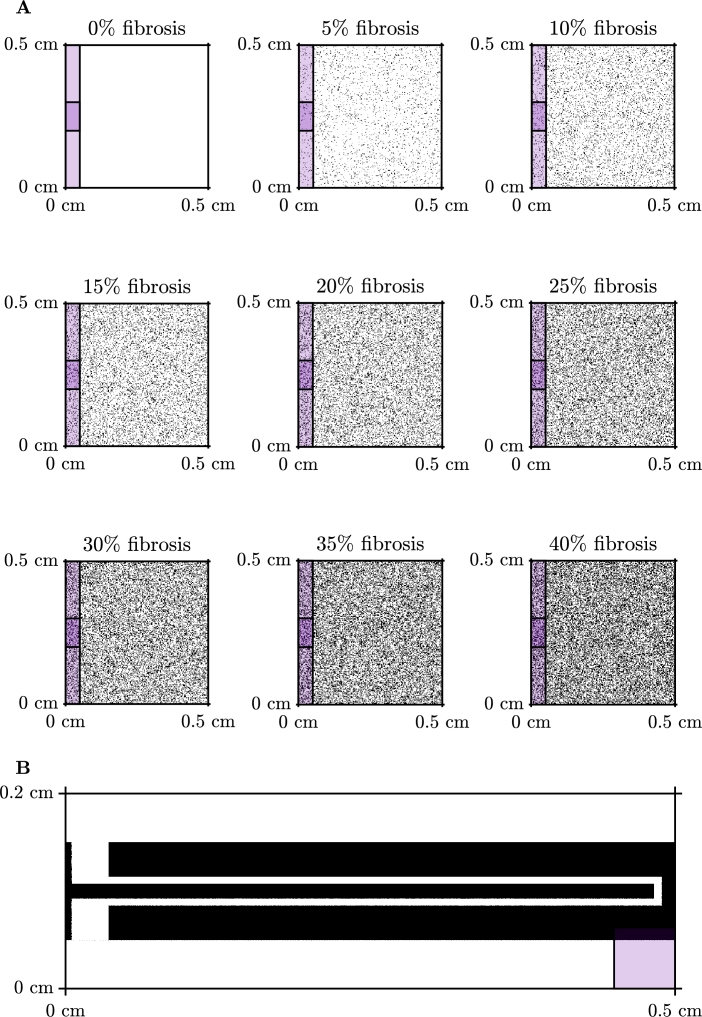
Domains used for the test problems presented in this section. Stimulus locations are indicated by the coloured overlay. **A**: Example of diffuse domains generated using a uniform random distribution for the isotropic and anisotropic test problems. The stimulus for the isotropic problem is indicated by the lighter purple region, and the stimulus for the anisotropic problem is indicated by the darker purple region. **B**: Tunnel domain used for the third test problem. (For interpretation of the colours in the figure(s), the reader is referred to the web version of this article.)

In this section, we present five numerical test problems to show the effectiveness of our approach to compute activation maps. We perform these tests on two types of domains, a square domain with varying levels of fibrosis as presented in Fig. 3A and a tunnel-like domain, as presented in Fig. 3B. We test the square domain for both isotropic and anisotropic conduction and isotropic conduction only in the tunnel domain. We include two further test cases in this section, these include a scar problem, where we compute the activation map for propagation through a scar region with varying amounts of fibrosis and varying conductivity, and propagation in a system that generates spiral waves.

For all of our numerical tests we use the monodomain model as presented in Section 3.1 and compute the activation time of the tissue, i.e., the time after the stimulus is applied that it takes for the membrane potential to become higher than a certain threshold, $V_{thresh}$. The parameters we have used in these tests are presented in Table 1. For the isotropic problems, we use the identity matrix for the conductivity tensor, Σ=ImS/cm, and we use

(13) $$\Sigma =\left[\begin{array}{cc} 0.625 & -0.375\\ -0.375 & 0.625 \end{array}\right]\;\text{mS/cm}$$

for the anisotropic problems, which is 4 times as conductive in the $-45^{\circ }$ direction than in the $45^{\circ }$ direction. For the isotropic problem we apply the stimulus to the left side of the domain $[0,0.05]\times [0,0.5]\;{\text{cm}}^{2}$, while for the anisotropic problem we apply the stimulus to a region on the left side of the domain $[0,0.05]\times [0.2,0.3]\;{\text{cm}}^{2}$ and for the tunnel problems we apply the stimulus to the bottom left corner $[0.48,0.5]\times [0,0.02]\;{\text{cm}}^{2}$.

We measure the error of the graph-based homogenisation methods by the square root of the mean squared relative error,

(14) $$\mathrm{error}=\sqrt{\text{mean}\left({\left(\frac{AT(x_{red})-AT^{⁎}(x_{red})}{AT(x_{red})}\right)}^{2}\right)}\times 100\text{\%}$$

where $x_{red}$ is the location of the reduced set of nodes, *AT* represents the activation times calculated for the reference solution using an activation threshold of $V_{thresh}=-40\;\text{mV}$ and $AT^{⁎}$ is the activation times calculated using the graph-based homogenisation approach.

### 4.1 Isotropic problem

**Fig. 4:**
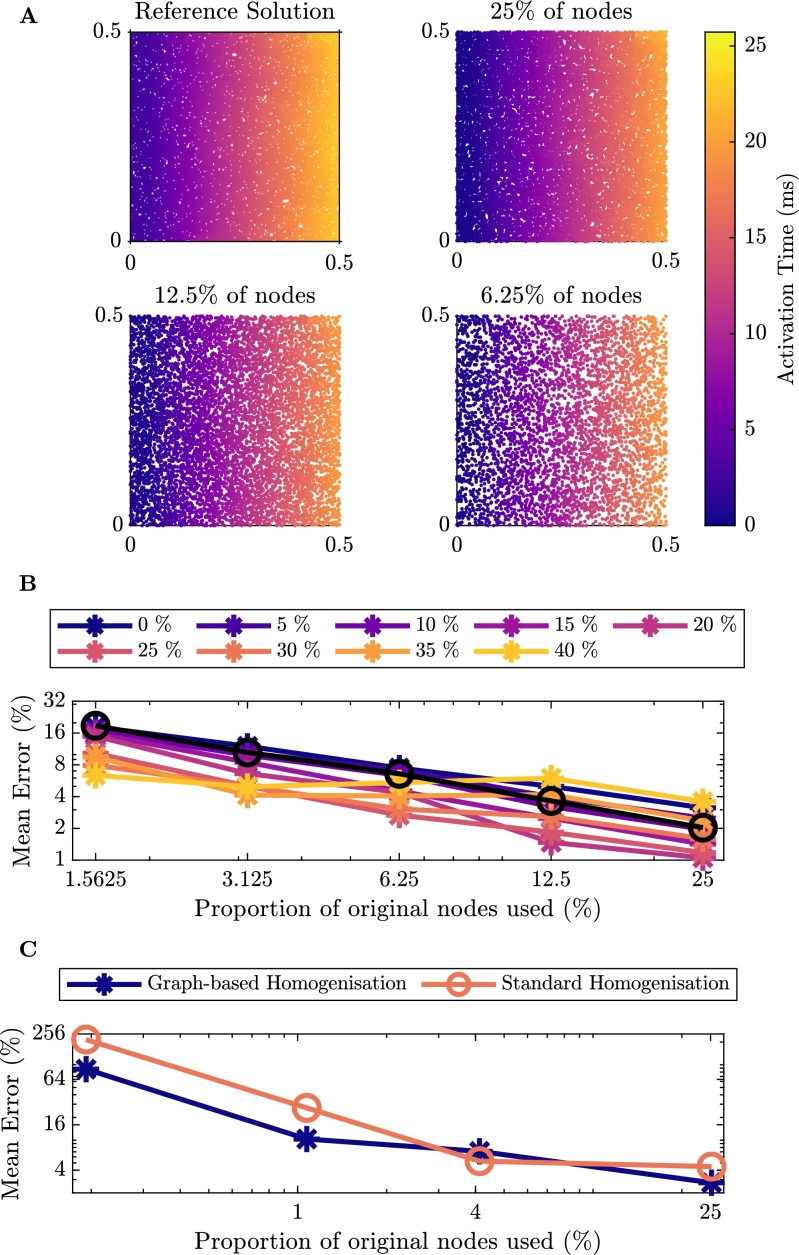
Results for isotropic conduction in tissue. **A**: Comparison of the activation maps of the graph-based homogenisation technique with varying reduction in the number of nodes in the mesh with 40% Fibrosis. **B**: Percentage error of activation times for the graph-based homogenisation method for the different levels of fibrosis. The black line on the figure represents the error associated with reducing the number of nodes in the mesh for the homogeneous problem. **C**: Percentage error of activation times comparing the graph-based homogenisation approach to the standard homogenisation approach with 40% fibrosis.

**Fig. A.10:**
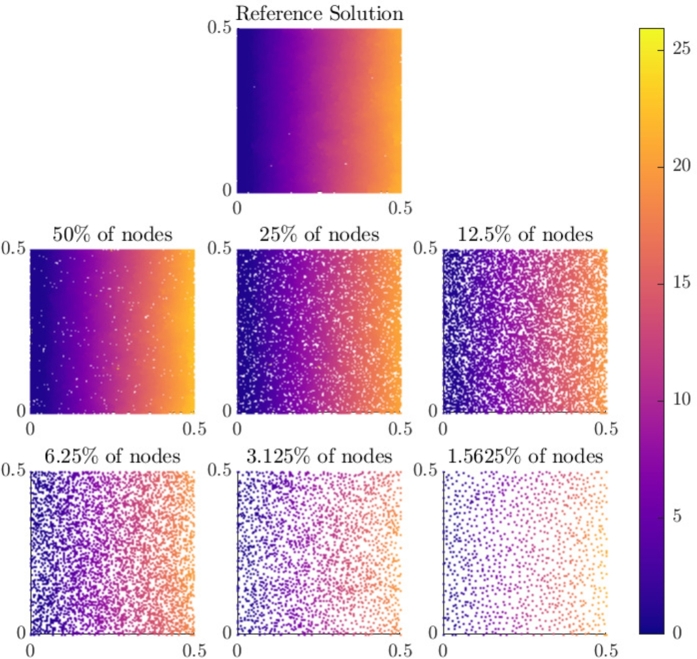
Comparison of the activation maps of the graph-based homogenisation technique with varying reduction in the number of nodes in the mesh for isotropic conduction in tissue with 40% fibrosis.

For the first set of test problems we consider isotropic propagation of an electrical signal across a two-dimensional square domain with varying levels of fibrosis. Due to qualitative similarities between each of the activation maps, we only show a sample of the activation maps for 40% fibrosis (Fig. 4A). The remaining activation maps for smaller amounts of nodes can be seen in the Appendix in Fig. A.10. In this scenario, activation propagates as a planar wavefront, but with some blurring due to the fibrosis. As the number of nodes in the homogenised problem decreases, the same behaviour is captured and graph-based homogenisation appears to perform well.

Fig. 4B shows the quantitative performance of the method on this problem, according to the error measure (14). A trade-off between the computational savings that come from reduced numbers of nodes and the error incurred is clearly seen, but with low errors still achievable for reductions in node count by an order of magnitude. In order to prove a better sense of the source of this error, Fig. 4B also displays the error associated with reducing the number of nodes in the homogeneous problem (black line). We observe here that for fibrosis of less than 30%, the error for each of the levels aligns well with the error for the homogeneous problem, decreasing linearly in the log-log plane. For fibrosis of 30% or more, smaller numbers of nodes actually achieve lower errors than the homogeneous case, and there is deviation from a linear decrease in the log-log plane. Regardless, the large errors also seen for the homogeneous problem show that the majority of error at low nodecounts arises from the numerical effect of changing the size of mesh elements, and not the ability of our graph-based homogenisation to represent the impact of the fibrosis.

In Fig. 4C, we present a comparison of the errors of the two homogenisation approaches for the isotropic problem with 40% fibrosis. We see for this problem that the graph-based homogenisation produces a smaller error for all but one of the reduction in node counts, where the two methods appear to perform equivalently.

### 4.2 Anisotropic problem

**Fig. 5:**
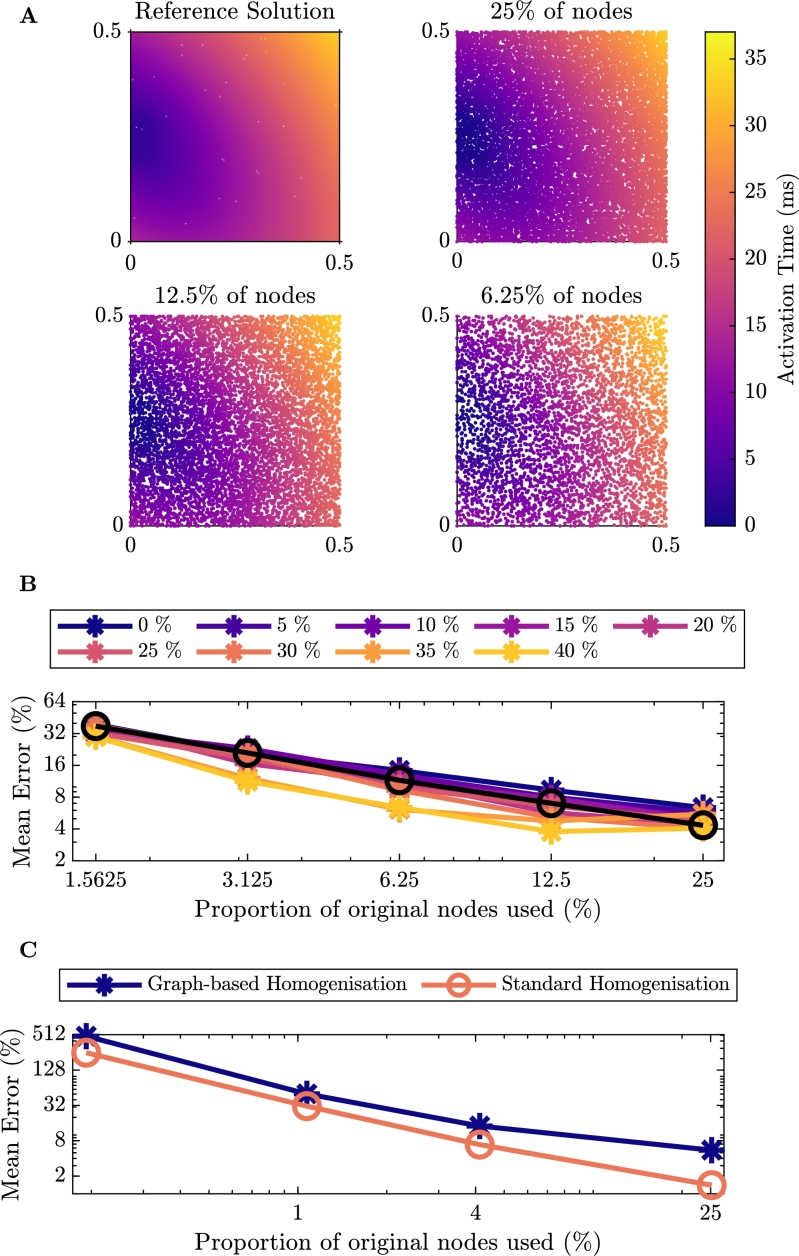
Results for anisotropic conduction in tissue. **A**: Comparison of the activation maps of the graph-based homogenisation technique with varying reduction in the number of nodes in the mesh with 15% Fibrosis. **B**: Percentage error of activation times for the graph-based homogenisation method for the different levels of fibrosis. The black line on the figure represents the error associated with reducing the number of nodes in the mesh for the homogeneous problem. **C**: Percentage error of activation times comparing the graph-based homogenisation approach to the standard homogenisation approach with 15% fibrosis.

**Fig. A.11:**
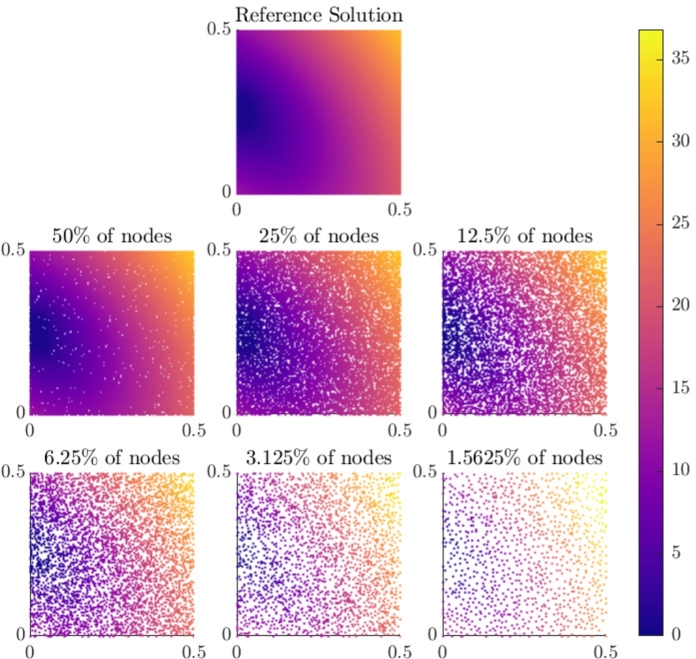
Comparison of the activation maps of the graph-based homogenisation technique with varying reduction in the number of nodes in the mesh for anisotropic conduction in tissue with 15% fibrosis.

For the second set of test problems we consider anisotropic propagation with conductivity defined as in equation (13). Here we consider the tissue to be four times less conductive from the bottom left of the tissue to the top right, at an angle of $45^{\circ }$. As with the isotropic problem, we consider a range of fibrosis from 0% to 40%, but due to qualitative similarities we only depict a sample of the simulation results for 15% fibrosis (Fig. 5A). The remaining activation maps for smaller numbers of nodes can be found in the Appendix in Fig. A.11. Graph-based homogenisation is again seen to perform well, as the Eikonal approach defining time costs (4) naturally incorporates anisotropies in conductivity.

Fig. 5B provides quantitative measurements of the error of the graph-based homogenisation for different levels of fibrosis and reductions in nodecount. Comparing with the error for the isotropic case (Fig. 4B), we see that in general, the error for the anisotropic problem is higher. Again however, performance is comparable with the corresponding homogeneous problem (black line), indicating that this error derives from the changes in mesh size and not homogenisation error.

In Fig. 5C, we present a comparison of the errors of the two homogenisation approaches for the anisotropic problem with 15% fibrosis. The scenarios of fibrosis that we tested here are generated in a homogeneous manner, and so for smaller levels of fibrosis, the assumption of periodicity on which the standard homogenisation methods are based is appropriate. As such, the weaknesses in standard homogenisation do not emerge. So, unlike in the isotropic case with 40% fibrosis, the standard homogenisation approach consistently performs better than the graph-based homogenisation.

### 4.3 Tunnel case

Our final test case is one that showcases the performance of the graph-based homogenisation. As we saw in Sections 4.1 and 4.2, both the graph-based homogenisation and the standard homogenisation methods perform well on a square of tissue with fibrosis. We designed the final test problem to be a domain that standard homogenisation methods will struggle with, that is, propagation through a thin channel. This domain, as depicted in Fig. 3B, is a tunnel structure connecting two regions of tissue, both with and without fibrosis. We apply the stimulus to the bottom right corner of the domain that propagates through the tunnel emerging at the top right of the domain. This domain was chosen as it reflects the zig-zag paths that can be found in some fibrotic tissue and is related to micro-reentry [58].

**Fig. 6:**
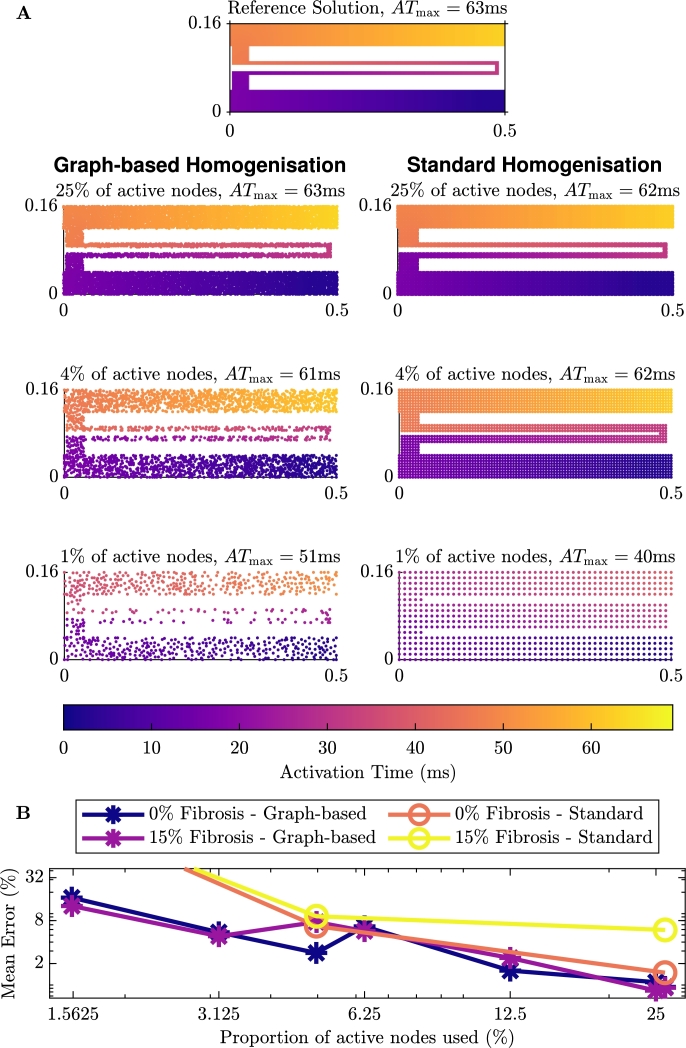
Comparison of the homogenisation techniques on the tunnel domain with no additional fibrosis. **A**: The left gives the graph-based homogenisation activation maps and the right gives standard homogenisation activation maps with the same number of nodes. **B**: Error associated with the homogenisation methods.

**Fig. A.12:**
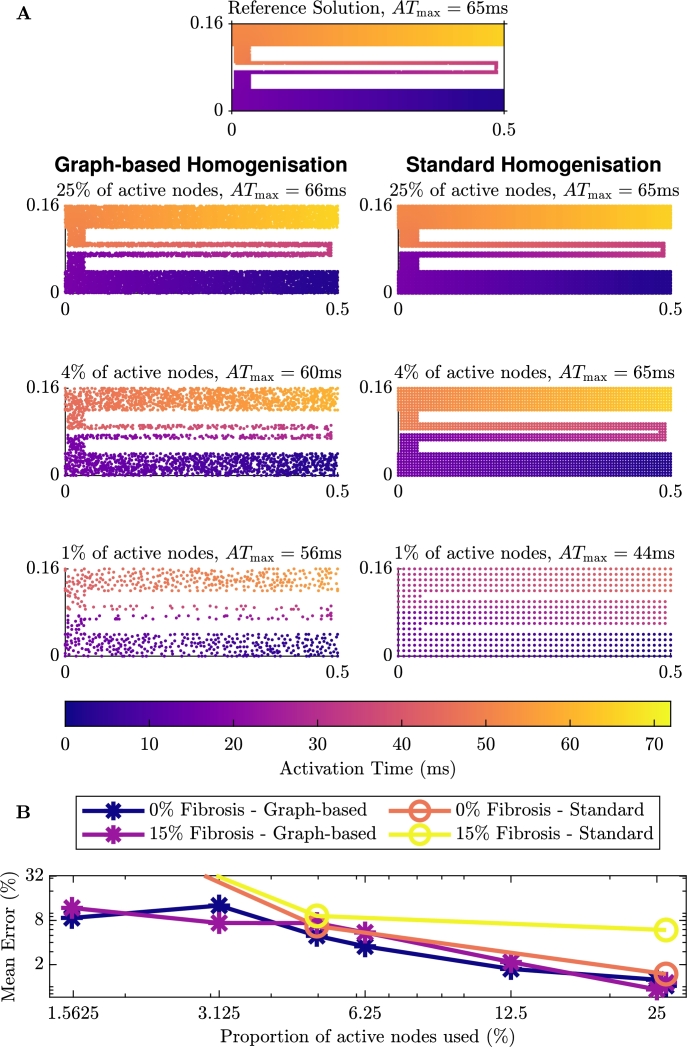
Comparison of the activation maps of homogenisation for the tunnel domain with 15% additional fibrosis. On the left if using the graph-based homogenisation techniques and on the right is standard homogenisation with the same number of nodes.

The activation maps on this domain can be seen in Fig. 6A (and Fig. A.12 in the Appendix for the case with additional fibrosis in the tunnel). We can see in the figures on the left that, as we would expect, each of the activation maps shows the propagation of the signal from the point of stimulus, through the tunnel until the end of the tunnel. For each of the reduced numbers of nodes, the graph-based homogenisation approach performs well. Comparing the maximum activation time for the problem without (63 ms) and with 15% additional fibrosis (66 ms), we also see that the addition of the fibrosis slows the propagation of the signal and as such for the case with additional fibrosis, the time to reach the end of the tunnel is higher. In the figures on the right, we see the results of standard homogenisation techniques (using the homogenisation technique outlined in Section 3.4) for the same problem with 25%, 4% and 1% of the active nodes. We see here, that while the standard homogenisation appears to perform quite well for 25% and 4% of the nodes, for 1% of the nodes the electrical signal leaks across the channel, causing premature excitation in the tissue beyond the tunnel.

As with our previous test problems, we measure the quantitative error using equation (14). We have depicted the error for this test problem in Fig. 6B. We see that for the case with no additional fibrosis, the error decreases when more nodes are used in the macroscale mesh. Also depicted in Fig. 6B is the error for the standard homogenisation on test problems shown in Fig. 6A. This shows that for homogenisation with approximately 4% and 25% of the original nodes, both with and without additional fibrosis, the error in the standard homogenisation and the graph-based homogenisation are of the same order, with the error from the graph-based homogenisation being lower. As we noted earlier, the standard homogenisation with approximately 1% of the original nodes has leakage across the channel, and as such, the error reflects this.

### 4.4 Scar problem

**Fig. 7:**
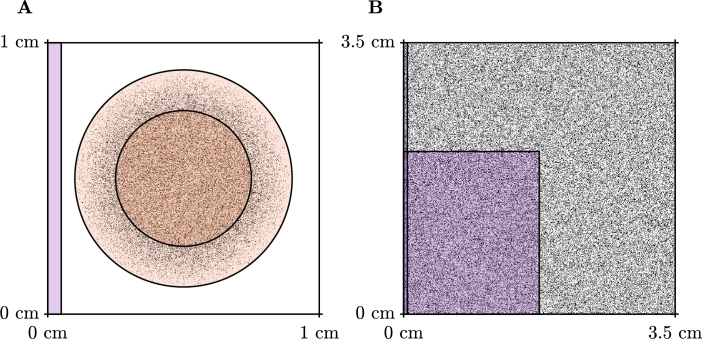
Simulation setups for the scar (**A**) and spiral wave (**B**) test problems. The orange (pale orange) region depicts the scar region (border zone), which exhibits non-conductive fibrotic elements (black pixels) and decreased conductivity. Purple regions indicate stimulus locations. For the spiral wave setup, self-sustained excitation is initiated by appropriately timed stimuli in the cross field stimulus configuration, as pictured.

Similar to other studies (for example [59]), we represent scarring as a circular region (radius 0.25 cm) in which replacement fibrosis has resulted in reduced conductivity and the presence of wholly non-conductive elements. Surrounding this region is a border zone, where the degree of fibrosis decreases gradually outwards to the edge of the border zone (radius 0.4 cm). Fibrotic obstructions occupy 30% of the elements in the scar region, decreasing linearly to 0% through the border zone. Reduced conductivity is incorporated by setting element conductivities to

(15) $$\Sigma =c_{f}\left[\begin{array}{cc} 3 & 0\\ 0 & 1 \end{array}\right]\;\text{mS/cm},$$

where $c_{f}$ is a connectivity factor that is equal to 1 outside the scar, 0.5 in the scar and changes linearly between these two values through the border zone. This also makes this problem a test of the ability of our homogenisation to handle variation in the diffusivity within single macroscopic elements. The domain is shown in Fig. 7A.

**Fig. 8:**
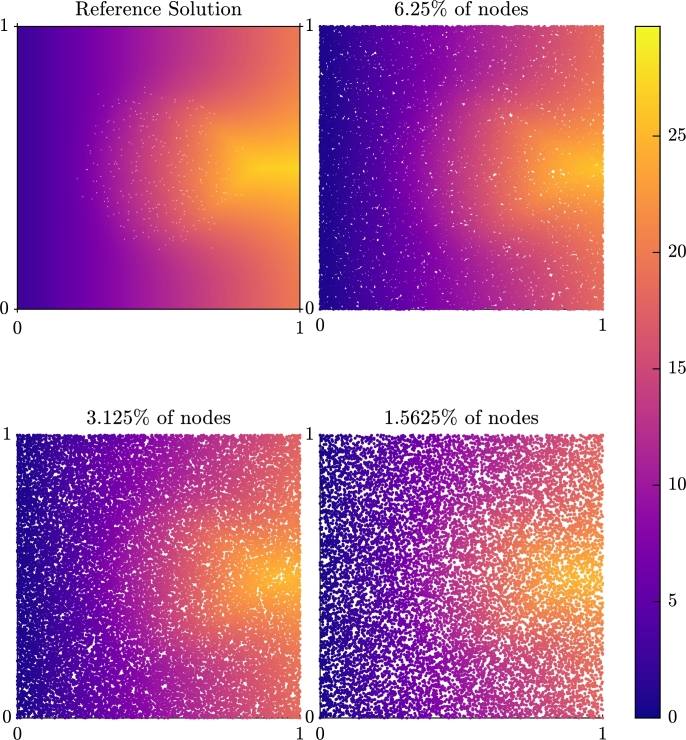
Comparison of the activation time (in mV) for a simulation through a scar region with 6.25%, 3.125% and 1.5625% of the nodes against the micro-scale reference solution.

Fig. 8 depicts the activation map for this problem, when a stimulus is applied on the left side of the domain. Activation is seen to propagate rapidly around the scar region, but the slower rate of conduction in the vertical direction results in latest activation times occurring behind the scar region. This activation delay is well recovered by the reduced-node models produced by graph homogenisation, in terms of both time and pattern of activation. The only significant effect of reducing the number of nodes to the extents tested (by more than 50 times) is a blurring of the precise “winged” shape of activation around the back edge of the scar.

### 4.5 Spiral wave problem

As a key phenomenon driving arrhythmia, it is important that homogenised models faithfully represent the dynamics of spiral wave re-entry. We test this by initiating spiral waves using a cross field stimulus pattern, in two-dimensional slices of tissue occupied by diffuse fibrosis, as represented by randomly-placed non-conductive fibrotic elements (Fig. 7B). In accordance with cardiac tissue, we use an anisotropic conduction tensor as in equation (15), with $c_{f}=1$ throughout the whole domain in this case. In contrast to the previous test cases, the cell model used for this problem is the Courtemanche–Ramirez–Nattel model [60], modified for chronic atrial fibrillation conditions according to the parameter changes compiled in [61] ($I_{Na}↓10\text{\%}$, $I_{to}↓80\text{\%}$, $I_{Kur}↓50\text{\%}$, $I_{K1}↑100\text{\%}$, $I_{Ca,L}↓40\text{\%}$, $g_{NaCa}↑40\text{\%}$, where changes indicated are relative to the published parameter values from [60]). This makes the study both more relevant to atrial arrhythmia conditions, and reduces the size of the domain required to support spiral waves.

**Fig. 9:**
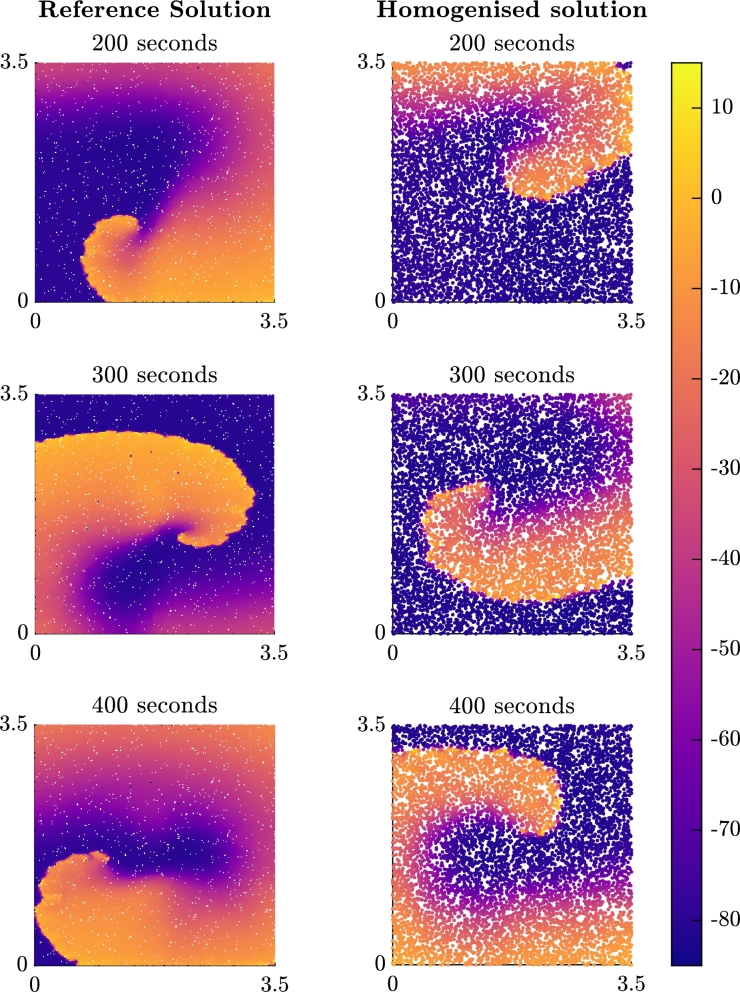
Plots of the membrane potential (in mV) for the spiral problem at snapshots in time after the second stimulus is applied. The title of each subfigure represents the time since the second stimulus in the cross field stimulus protocol that initiates the re-entrant pattern of activation.

The results for the spiral problem with 6.25% of the original nodes can be seen in Fig. 9. The homogenised model supports spiral waves that appear to be stable, but the path of the rotor tip does notably differ from the path observed in the finescale model. This does also manifest in differences in the dominant frequency of re-entry, at least during the period simulated in this text case. We expect that at least some of this error arises due to the sensitivity of cardiac simulations to the grid spacing, especially considering the larger spacings used in these simulations in order to accommodate sufficient tissue for a spiral wave pattern of re-entrant activation. However, it is also notable that the Eikonal (or FMM) approach does not inherently capture the complete effects of wavefront curvature on propagation speed. We return to this point in the Discussion.

## 5 Discussion

In Section 4, we presented a number of test problems to exhibit the effectiveness of the methods presented in Section 3.3. From these results, we can see that the methods can effectively deal with multiple different types of heterogeneous domains, diffuse (as shown in Fig. 3A) or structured (the tunnel in Fig. 3B).

These methods are shown to perform well in improving the efficiency of modelling spatial heterogeneities. The efficiency of these novel methods can be described in terms of the time taken to generate the reduced mesh, and the ability to calculate the effective diffusivities and to evaluate the monodomain solution. The computation times required are problem specific. For the anisotropic problem discussed in Section 4 with 15% fibrosis, the individual timings are shown in Table 2. Here we see the computation of the micro-scale model takes 449 seconds. With 25% of the nodes, the mesh reduction takes 19 seconds. In addition, the computation of the effective diffusivities takes 84 seconds, and the homogenised monodomain simulation then takes 101 seconds for an overall computation time of 204 seconds. As expected, the best improvement in computation times is seen for the reduction to only 1.5675% ($2^{-6}$) of the nodes, with the overall computation time and monodomain computation time being only 70 seconds and 10 seconds, respectively. We see an overall speed up of 6.4 and a speed up of more than 46 for the monodomain simulation.

**Table 2:** Computation time of our graph-based homogenisation method for the anisotropic problem with 15% fibrosis that is presented in Section 4.

		Proportion of original nodes				
	Micro-scale	2^−2^	2^−3^	2^−4^	2^−5^	2^−6^
Total time (s)	449	204	137	99	79	70
Monodomain time (s)	449	101	56	25	14	10
Mesh reduction (s)	-	19	11	7	4	3
Compute Σ_eff_ (s)	-	84	70	67	60	57

Monodomain speed up	-	4.5	8.0	18.2	31.8	46.3
Overall speed up	-	2.2	3.3	4.5	5.7	6.4

It is also important to note that the mesh generation and computation of the effective diffusivity need only be performed once for a given domain. So simulations could be run for problems with different cell models, stimulus protocols and model parameters without needing to repeat the mesh generation and homogenisation processes. For the anisotropic problem with 15% fibrosis and the parameters presented in Section 4, this means that to rerun the simulation using $2^{-6}$ of the nodes would take only 10 seconds, rather than the 449 seconds that the micro-scale simulation takes. For longer simulations of cardiac activity, greater savings will be seen.

We present a comparison of the timings of the two homogenisation approaches in Table 3 for the test problem presented in Section 4.1 with 40% fibrosis (the timings for the anisotropic problem with 15% fibrosis, are shown in the Appendix in Table B.4). In this table we see that the monodomain computation times for both the graph-based homogenisation and the standard homogenisation are similar, as they have the same number of nodes in the simulation, any differences come from the efficiency of computing the incomplete LU factorisation and solving the linear system with the stabilised biconjugate gradient method for the individual matrices. The standard homogenisation approaches are faster at computing the effective diffusivities, however, we reiterate that this represents a one-off cost and there is also a trade-off to be made in the computational error, as was shown in Fig. 4C. We also reiterate that there are certain scenarios in which standard homogenisation techniques fail, such as the tunnel domain presented in Section 4.3. The graph-based methods provide a good robust alternative to standard homogenisation in these situations.

**Table 3:** Comparison of computation times of our graph-based homogenisation method against standard homogenisation for the isotropic problem with 40% fibrosis that is presented in Section 4.

	Proportion of original nodes			
	2^−2^	5^−2^	10^−2^	25^−2^
**Graph-based homogenisation**				
Total time (s)	144	64	48	44
Monodomain time (s)	52	9	4	3
Compute Σ_eff_ (s)	72	50	42	39

Monodomain speed up	4.5	27.2	63.1	82.7
Overall speed up	1.6	3.7	4.9	5.3

**Standard homogenisation**				
Total time (s)	66	13	7	8
Monodomain time (s)	54	9	5	6
Compute Σ_eff_ (s)	12	3	2	2

Monodomain speed up	4.4	24.6	45.8	37.7
Overall speed up	3.5	18.7	33.5	28.9

The results show that the graph-based homogenisation methods do not introduce any significant error. The error in reducing the number of nodes in the graph-based process align with the error in a reduction of nodes for the homogeneous problem. A further avenue for reducing the error in the calculation of the effective diffusivities could be to consider higher order FMM methods [62], however, the FMM method used in this work appears to perform well and so adding the extra computational cost seems unnecessary. We note that for the spiral problem, although the homogenised model is able to produce spiral waves, they do not exhibit the correct frequency of rotation. We believe this is due to numerical effects of gridsize, but could also potentially have arisen due to the base Eikonal method not properly taking into account curvature effects. An avenue of future work is to investigate extended graph-based methods for activation time that are better able to capture the effects of wavefront curvature [63], [64]. This would not change the underlying idea of our graph-based homogenisation, simply the method used to calculate the communication times that are used to derive effective conductivities. However, as the homogenisation process is conducted separate to the simulation process, such efforts to explicitly incorporate curvature would need to pre-specify the nature of this curvature.

The methods presented in this work can be extended to three dimensions. The quality measure for the reduction in mesh can be adapted to incorporate a measure for tetrahedral quality, rather than triangle quality. The graph based homogenisation can also be adapted to three dimensions, with the three dimensional version of FMM. However, in this approach, there is the added complication of allowing paths from anywhere on a face of an element, rather than an edge. The extension of the graph-based homogenisation methods to three-dimensions is possible and implementable but careful consideration is required to ensure computational efficiency.

An area of interest in cardiac modelling that is related to modelling fibrosis, is that of modelling scar tissue [58], [65]. In this area, there are often scenarios where propagation occurs in channels through the scar similarly to the tunnel case presented in Section 4.3. The effectiveness of the graph-based homogenisation in the thin channel example and in problems with high levels of fibrosis leads us to believe these methods would be particularly significant in scar modelling.

The graph-based homogenisation approach could be improved to allow the macro-scale mesh to consist of any point within healthy tissue, rather than constrained to the micro-scale node positions. Implementing this approach would increase the number of nodes in the graph, but could be implemented using equation (3) and connecting the off-grid node to the corners of the micro-scale element it is contained in. The methods could also be adapted to using a quadrilateral macroscale mesh. This involves using a least squares approach to solving the four or six term equivalent of equation (8) for the effective diffusivities on the quadrilateral elements. With these improvements, better quality meshes could be obtained and used for the PDE simulation.

## 6 Conclusions

We have presented a novel numerical method for upscaling heterogeneous domains that makes use of graph based techniques. With this method we are able to capture fine scale behaviour on a coarse scale mesh. This method has been shown to work on a number of different patterns of fibrotic heterogeneity, both diffuse and structured. This includes both isotropically and anisotropically conductive tissue, as well as tissue possessing spatially heterogeneous conductive properties. Observation of numerical errors in the majority of test cases shows that the graph-based homogenisation is not a significant source of error. However, using homogenisation to derive reduced-node models does also increase the grid spacing, which can increase traditional numerical error.

The methods presented in this paper lead to a promising new avenue for numerical upscaling in many different applications. It has been shown to be effective here for capturing fibrosis in cardiac electrophysiology. An interesting avenue for future investigation is to consider applications in other areas, such as any problem with travelling wave dynamics where considering time costs for activation is reasonable. An extension of these methods into three dimensions in order to model more anatomically-correct domains of cardiac tissue will be considered in later work.

## CRediT authorship contribution statement

**Megan E. Farquhar:** Conceptualization, Formal analysis, Methodology, Software, Visualization, Writing – original draft, Writing – review & editing. **Kevin Burrage:** Conceptualization, Funding acquisition, Methodology, Supervision, Writing – review & editing. **Rodrigo Weber Dos Santos:** Supervision, Writing – review & editing. **Alfonso Bueno-Orovio:** Supervision, Writing – review & editing. **Brodie A.J. Lawson:** Conceptualization, Formal analysis, Methodology, Software, Writing – original draft, Writing – review & editing.

## Declaration of Competing Interest

The authors declare that they have no known competing financial interests or personal relationships that could have appeared to influence the work reported in this paper.

## Figures and Tables

**Table B.4:** Comparison of computation times of our graph-based homogenisation method against standard homogenisation for the anisotropic problem with 15% fibrosis that are presented in Section 4.

	Proportion of original nodes			
	2^−2^	5^−2^	10^−2^	25^−2^
**Graph-based homogenisation**				
Total time (s)	222	86	68	65
Monodomain time (s)	108	16	8	10
Compute Σ_eff_ (s)	91	64	57	53

Monodomain speed up	3.2	21.5	42.2	34.9
Overall speed up	1.6	4.1	5.2	5.4

**Standard homogenisation**				
Total time (s)	90	17	9	12
Monodomain time (s)	81	13	7	8
Compute Σ_eff_ (s)	10	3	2	3

Monodomain speed up	4.3	26.5	50.6	42.4
Overall speed up	3.9	21.1	37.5	30.0

## Appendix A Additional figures

See Fig. A.10, Fig. A.11, Fig. A.12 below.
